# Italian Validation of the Patient Satisfaction With Nursing Care Quality Questionnaire (PSNCQQ): A Multicenter Psychometric Study

**DOI:** 10.1155/jonm/7681817

**Published:** 2026-10-08

**Authors:** Donatella D’Accolti, Paolo Iovino, Cinzia Anna Maria Papappicco, Sipontina Zerulo, Federico Ruta

**Affiliations:** ^1^ AOUC Policlinic of Bari Bari Italy; ^2^ Department of Health Sciences University of Florence Florence Italy unifi.it; ^3^ ASL Bari San Paolo Hospital Bari Italy ao-sanpaolo.it; ^4^ Department of Medical and Surgical Sciences University of Foggia Foggia Italy unifg.it; ^5^ Health Agency BAT, General Direction Andria Province of Barletta-Andria-Trani Italy

**Keywords:** factor analysis, nursing care, patient-reported outcomes, patient satisfaction, PSNCQQ, psychometrics, quality of healthcare, reliability, statistical

## Abstract

**Background:**

The Patient Satisfaction with Nursing Care Quality Questionnaire (PSNCQQ) is one of the most widely used international instruments for assessing patient‐perceived nursing care quality. However, no validated Italian version is currently available.

**Aim:**

To evaluate the psychometric properties of the Italian version of the PSNCQQ in a large sample of hospitalized adult patients.

**Methods:**

A multicenter cross‐sectional study was conducted in five public hospitals in Italy. The PSNCQQ was translated and culturally adapted according to international guidelines. The total sample (*N* = 698) was randomly divided into two independent subsamples for exploratory factor analysis and confirmatory factor analysis. Models were estimated using robust weighted least squares with adjustment for hospital clustering. Competing factorial models were tested. Internal consistency was assessed using Cronbach’s alpha and McDonald’s omega, whereas convergent and construct validity were examined through average variance extracted, composite reliability, and correlations with four global satisfaction indicators.

**Results:**

Exploratory factor analysis supported a two‐factor structure distinguishing core nursing care, comprising 14 items, from continuity‐of‐care and discharge‐related processes, comprising five items. Confirmatory factor analysis showed that the correlated two‐factor model provided a better fit than the strictly unidimensional model, whereas the bifactor model was not retained. Internal consistency was excellent for core nursing care (*ω* = 0.964), continuity of care (*ω* = 0.894), and the total scale (*α* = 0.95; *ω* = 0.95). Average variance extracted and composite reliability values supported convergent validity. PSNCQQ scores were positively associated with global satisfaction indicators (*r* = 0.45–0.88; *p* < 0.001), with the strongest associations observed for the core nursing care dimension.

**Conclusions:**

In the Italian context, patient satisfaction with nursing care is best represented by two related but distinct domains. The Italian PSNCQQ demonstrated robust measurement properties and supports the interpretation of both subscale and total scores for evaluating patient experience in hospital settings.

**Implications for Nursing Management:**

The PSNCQQ can provide nurse managers with a reliable patient‐reported indicator for monitoring nursing‐sensitive quality across hospital units. Its separate assessment of core nursing care and continuity‐related processes may support benchmarking, quality‐improvement planning, and targeted organizational interventions in bedside care, discharge preparation, and care coordination.

## 1. Background

Access to high‐quality nursing care is a fundamental human right and a cornerstone for ensuring positive patient outcomes [1]. Due to the intensity and continuity of their interactions with patients, nurses play an essential and irreplaceable role within healthcare systems [2]. Over time, the nursing profession has redefined and innovated care delivery models to enhance patient satisfaction [3], which is widely recognized as the primary indicator of nursing care quality [4].

Assessing the quality of nursing care is particularly significant, as nurses constitute the largest group of healthcare professionals [5] and the quality of their performance is a critical metric for evaluating the overall efficiency and effectiveness of healthcare systems. Furthermore, the nursing role encompasses the collection, analysis, and interpretation of data related to patients’ health needs, with nurses assuming primary responsibility for implementing targeted interventions aimed at harm prevention and the enhancement of care safety and quality. Improving care quality not only leads to better clinical outcomes but also contributes to reducing healthcare costs [6].

Although nursing is a practice‐oriented discipline, it involves continuous interaction with individuals and families, necessitating the development of transversal competencies—commonly referred to as soft skills—such as critical thinking, effective communication, and teamwork [7]. Numerous studies have demonstrated that a high level of soft skills is positively associated with both healthcare outcomes and patient satisfaction [7–9].

Patient satisfaction with nursing care quality is one of the strongest predictors of overall satisfaction with healthcare services [10]. As a subjective construct, satisfaction reflects patients’ personal perceptions and emotions, even when healthcare professionals may hold differing views. Several studies have identified key determinants of satisfaction, including healthcare staff attitude and competence, accessibility of care, effectiveness of clinical responses [11–13], and communication between healthcare providers and patients [14, 15].

High‐quality nursing care is associated with lower readmission rates, faster recovery, and increased patient safety throughout the care process [16]. Analyzing the perceptions and factors influencing patient satisfaction with the quality of care and services received during hospitalization enables the identification of critical elements for designing targeted interventions aimed at improving care quality and safety, while also reducing hospital stays and associated costs [17, 18].

For a rigorous assessment of patient satisfaction with hospital nursing care, it is essential to employ valid and reliable instruments capable of providing robust evidence to support organizational and clinical decision‐making. Measuring patient satisfaction with nursing care quality can effectively enhance nursing practice by facilitating the development of care standards [19]. Among the most widely used and validated tools for assessing patient satisfaction with nursing care are the La Monica–Oberst Patient Satisfaction Scale (LOPSS) [20], the Newcastle Satisfaction with Nursing Scale (NSNS) [21], and the Patient Satisfaction with Nursing Care Quality Questionnaire (PSNCQQ) [22]. The PSNCQQ is the most frequently employed in international research and has demonstrated validity across multicultural settings and diverse healthcare systems [19].

Despite extensive international evidence, no studies have yet validated an Italian version of the PSNCQQ. There is a lack of data regarding its reliability and validity in the Italian context, its psychometric properties, and its application in local clinical settings. Exploring hospitalized patients’ perceptions and evaluations of nursing care quality will help identify satisfaction determinants in relation to individual patient characteristics. This will enable future research to adopt standardized and reliable instruments, thereby supporting decision‐makers and facilitating cross‐national comparisons. Therefore, the aim of this study was to evaluate the psychometric properties of the Italian version of the PSNCQQ in a large sample of hospitalized adult patients.

## 2. Methods

### 2.1. Study Design and Setting

A cross‐sectional multicenter study was conducted between July 2023 and July 2024 in five public hospitals located in the Apulia region in Italy. The study involved medical and surgical wards providing inpatient care. Data were collected using a structured questionnaire administered during hospitalization or within 48 h prior to discharge. For transparency, the methodological procedures adopted in the present validation study were mapped against the applicable COSMIN standards. The completed assessment, including ratings and supporting justifications, is provided in Supporting Information S1.

### 2.2. Participants and Procedure

Eligible participants were adult inpatients (≥ 18 years), hospitalized for at least 48 h, able to communicate in Italian, and cognitively nonimpaired. Exclusion criteria were diagnosis of cognitive impairment (Mini‐Mental State Examination [MMSE] score ≤ 25 [23]), severe clinical instability, end‐of‐life palliative care, admission to acute psychiatric settings (only medical and surgical wards were included), and inability or refusal to provide informed consent. Patients with signs of acute confusion or delirium were excluded using the confusion assessment method (CAM) [24], which identifies delirium based on the presence of Features 1 (acute onset and fluctuating course) and 2 (inattention), plus either Feature 3 (disorganized thinking) or 4 (altered level of consciousness). Delirium was screened to ensure valid and reliable responses to patient‐reported outcomes, as cognitive fluctuations may compromise response accuracy. Participants were recruited consecutively by trained research nurses who explained the study objectives and procedures. After obtaining written consent, anonymized questionnaires were completed independently by patients or, if required, with minimal assistance from trained staff to prevent response bias.

### 2.3. Instrument

The PSNCQQ is a 19‐item instrument developed by Laschinger et al. [22] to measure patients’ perceptions of nursing care quality during hospitalization. Items are rated on a 5‐point Likert scale (1 = *poor* to 5 = *excellent*), covering dimensions such as information and communication, responsiveness, professionalism, empathy, privacy, continuity, and discharge planning. Four additional global items assess perceived overall hospital care quality, overall nursing care quality, self‐rated health, and willingness to recommend the hospital. Higher scores indicate higher satisfaction.

### 2.4. Translation and Cultural Adaptation

The PSNCQQ was translated into Italian following international guidelines for cross‐cultural adaptation of patient‐reported outcome measures [25]. The process included the following: (i) forward translation by two independent bilingual translators; (ii) synthesis and reconciliation of translations; (iii) back‐translation by two native English speakers blinded to the original version; (iv) expert panel review (*n* = 7 nurses and researchers in healthcare quality) for semantic, conceptual, and cultural equivalence; (v) cognitive debriefing with 15 hospitalized patients to assess clarity, comprehensibility, and relevance of items; and (vi) final proofreading and pretesting. Permission to translate, adapt, and validate the PSNCQQ into Italian was obtained from the original author prior to the start of the study.

Following the synthesis and reconciliation of the translated versions, content validity was assessed by an expert panel composed of seven senior nurses and nursing professors with expertise in clinical governance, nursing management, and patient experience measurement. The experts independently evaluated semantic, conceptual, and cultural equivalence of each item and assessed relevance using a structured grid based on the Content Validity Index (CVI) criteria. During this process, Item 5 (“Informing family or friends”) was modified to improve cultural appropriateness and legal coherence with privacy and professional regulations. The experts noted that direct communication with family members about a patient’s condition without explicit consent is restricted by the European Union General Data Protection Regulation (GDPR; Regulation (EU) 2016/679) and by the Italian Nurses’ Code of Ethics (Art. 19, Italian National Federation of Nursing Professions [FNOPI], 2019), which emphasizes confidentiality and protection of sensitive health information. Therefore, the item was reformulated to shift the focus from unilateral information‐sharing to patient‐authorized involvement of family members in care planning, resulting in the following version: *“Coinvolgimento di familiari o amici nella pianificazione della tua assistenza”* (“Involvement of family or friends in your care planning”).

To assess face validity and comprehensibility, cognitive interviews were conducted with 15 hospitalized patients using the “think‐aloud” method and probing questions. Participants reported no difficulties in understanding the items or response options, and the revised Item 5 was judged clear, acceptable, and respectful of patient autonomy and privacy. No further changes were required.

### 2.5. Data Collection and Management

Data were collected using paper‐based questionnaires, which were subsequently entered into a secure electronic database by two independent operators to ensure accuracy. Missing data were monitored and handled using predefined rules. Participants’ sociodemographic and clinical characteristics were also recorded, including age, gender, marital status, education, employment status, number of hospitalizations, type of admission, room occupancy, perceived health status, and cognitive function.

### 2.6. Statistical Analysis

Descriptive statistics were computed to summarize participants’ sociodemographic characteristics, using means and standard deviations for continuous variables and frequencies and percentages for categorical variables. Item‐level characteristics of the PSNCQQ were examined by inspecting response distributions, total scale scores, and the four global evaluative items complementing the instrument.

Structural validity was assessed using a cross‐validation approach. The dataset was randomly split into two independent subsamples. Exploratory factor analysis (EFA) was conducted in the derivation sample to identify the underlying structure. Confirmatory factor analysis (CFA) was subsequently performed in the validation sample to test competing models. All 19 items were treated as ordered categorical indicators and analyzed using the robust weighted least squares mean‐ and variance‐adjusted (WLSMV) estimator. Because patients were nested within hospitals, models were estimated with the TYPE = COMPLEX option [26], specifying hospital as the clustering variable. Intraclass correlation coefficients (ICC[1]) were computed to quantify clustering effects; values ranging from 0.23 to 0.86 supported the use of cluster‐adjusted estimation.

Sample size adequacy was established a priori on the basis of recognized criteria for factor‐analytic studies rather than through a power calculation for hypothesis testing. For the EFA, a subject‐to‐item ratio of at least 10:1 and an absolute minimum of 300 respondents were adopted [27, 28]. For the CFA, the COnsensus‐based Standards for the selection of health Measurement INstruments (COSMIN) methodology rates a study as very good for structural validity when the sample includes at least seven respondents per item and no fewer than 100 respondents overall [29], while simulation studies indicate that samples of 200–300 yield stable parameter estimates for models with high communalities and standardized loadings above 0.70 [30, 31]. The total sample (*N* = 698) was randomly split into a derivation subsample (*n* = 349) and a validation subsample (*n* = 349); each corresponds to a subject‐to‐item ratio of approximately 18:1 and exceeds the threshold of 133 respondents implied by the COSMIN criterion for a 19‐item instrument. Both subsamples therefore met the requirements for the exploratory and the confirmatory analysis, and the split‐sample design ensured that the confirmatory model was first tested on data independent of those from which it was derived.

In the validation sample, three models were tested: (1) a unidimensional model reflecting the original theoretical structure [22]; (2) a correlated two‐factor model distinguishing core nursing care during hospitalization from continuity/discharge and family involvement; and (3) a bifactor model including a general satisfaction factor and an orthogonal specific continuity/discharge factor. Finally, a correlated two‐factor model was also tested in the whole sample to obtain more precise parameter estimates for the retained structure. Model fit was evaluated using *χ* ^2^, Comparative Fit Index (CFI ≥ 0.95), Tucker–Lewis Index (TLI ≥ 0.90), root mean square error of approximation (RMSEA ≤ 0.08), and standardized root mean square residual (SRMR ≤ 0.08) [32]. Standardized factor loadings ≥ |0.30| were considered acceptable [33].

Internal consistency of the retained model was assessed using Cronbach’s alpha and McDonald’s omega. Composite reliability (CR) and average variance extracted (AVE) were calculated to evaluate convergent validity. Values of AVE ≥ 0.50 and CR ≥ 0.70 were considered indicative of adequate convergent validity [34]. Corrected item–total correlations were examined to assess item discrimination (≥ 0.30 indicating adequate homogeneity).

Construct validity was verified by testing correlations between the total PSNCQQ score and the four additional items complementing the scale—overall perception of care quality, nursing care, health, and willingness to recommend the hospital to family and friends—under the hypothesis of positive and strong associations. Finally, the relationships between the total PSNCQQ score and age and gender were explored, hypothesizing that older patients would be more satisfied with nursing care [35], whereas female patients would be less satisfied than males [36, 37].

### 2.7. Ethical Considerations

The study adhered to the Declaration of Helsinki and was approved by the Independent Ethics Committee of University Hospital of Bari under protocol number 38977/20/04/23. Participation was voluntary, and confidentiality was guaranteed using anonymized codes. All participants provided written informed consent prior to participation.

## 3. Results

### 3.1. Participant Characteristics

Table 1 presents the sociodemographic and clinical characteristics of the participants (*N* = 698). The mean age was 62.8 years (SD = 15.2, range 22–95). Slightly more than half were men (51.6%), while 47.9% were women and 0.6% identified as nonbinary. Nearly all participants were Italian (99.4%). Most were married or cohabiting (70.1%), followed by widowed (13.5%) and single (12.0%) individuals. In terms of education, the vast majority held a high school diploma (85.5%), whereas 5.0% had a bachelor’s degree, 4.4% a master’s degree, and fewer than 1% had a first‐ or second‐level master’s or a PhD. Regarding occupational status, about two in five participants were employed (41.1%), while a comparable proportion were retired or unemployed (41.8%). Most hospitalizations were unplanned, with 61.7% of participants admitted through the emergency department, 21.1% for ordinary (scheduled) admissions, and 11.6% following a screening or procedure. Over half of the patients (51.9%) stayed in a double room, 38.4% in multibed rooms, and 9.7% in single rooms. The majority (58.0%) were hospitalized once, 23.4% twice, and 12.0% three times. Perceived health status was generally positive, with 51.1% rating their health as good and 28.7% as fair, while 14.5% reported poor or very poor health. Cognitive function was high, with a mean MMSE score of 28.6 (SD = 1.4, range 26–30).

**TABLE 1:** Sociodemographic and clinical characteristics of participants (*N* = 698).

Variable	Mean (SD) [range]	*n* (%)
Age (years)	62.80 (15.16) [22–95]	—
Gender	—	
Male		360 (51.6)
Female		334 (47.9)
Nonbinary		4 (0.6)
Nationality	—	
Italian		694 (99.4)
Non‐Italian		4 (0.6)
Marital status	—	
Single		84 (12.0)
Married/cohabiting		489 (70.1)
Divorced		31 (4.4)
Widowed		94 (13.5)
Education^1^	—	
High school diploma		597 (85.5)
Bachelor’s degree		35 (5.0)
Master’s degree		31 (4.4)
First‐level master		5 (0.7)
Second‐level master or PhD		2 (0.2)
Occupational status^2^	—	
Employed		287 (41.1)
Retired/unemployed		292 (41.8)
Type of admission	—	
Emergency		431 (61.7)
Ordinary (scheduled)		147 (21.1)
After screening/procedure		81 (11.6)
Transferred from another setting		34 (4.9)
Other		5 (0.7)
Room occupancy	—	
Alone		68 (9.7)
With another person		362 (51.9)
With > 2 persons		268 (38.4)
Number of hospitalizations^3^	—	
Once		405 (58.0)
Twice		163 (23.4)
Three times		84 (12.0)
Four times or more		10 (1.4)
Perceived health status^4^	—	
Excellent		39 (5.6)
Good		357 (51.1)
Fair		200 (28.7)
Poor/very poor		101 (14.5)
Cognitive function (Mini‐Mental State)	28.62 (1.35) [26–30]	—

*Note:* Percentages are based on valid responses.

^1^28 missing values (4.0%).

^2^119 missing values (17.0%).

^3^36 missing values (5.2%).

^4^1 missing value (0.1%).

### 3.2. Descriptive Statistics of PSNCQQ Items

Table 2 reports the distribution of responses for the 19 PSNCQQ items. Overall, participants expressed favorable evaluations of nursing care, with most responses falling in the good to very good range. The response pattern showed adequate variability, with most items displaying well‐distributed frequencies across the five Likert categories and limited clustering at the extremes, supporting good sensitivity of the scale. Marked floor effects (≥ 15%) were observed for items addressing discharge and postdischarge processes, specifically discharge instructions (60.9%) and coordination of care after discharge (62.3%), and to a lesser extent for family involvement (informing family or friends, 26.1%, involving family/friends in care, 26.3%). These findings highlight lower satisfaction with continuity of care and communication with family members. Conversely, ceiling effects (≥ 15%) were evident for items representing interpersonal and professional aspects of nursing care—such as concern and caring by nurses (27.2%), helpfulness (20.3%), skill and competence of nurses (18.8%), and coordination of care (16.8%)—reflecting consistently high satisfaction in these domains. For the remaining items, both floor and ceiling rates were below the 15% criterion, confirming satisfactory response dispersion and discriminatory capacity across the instrument. Table 3 presents the response distributions for the four global satisfaction items. Most participants rated the overall quality of care and services and overall quality of nursing care as *very good* or *excellent* (approximately 60%), indicating strong overall satisfaction. By contrast, ratings for overall health self‐assessment were more evenly distributed, while recommendation of the hospital to family and friends showed the highest proportion of *excellent* responses (25%), reflecting a favorable global evaluation of hospital care.

**TABLE 2:** Response distribution for the PSNCQQ items (*N* = 692–698).

Item	*n*	*Poor*, *n* (%)	*Fair*, *n* (%)	*Good*, *n* (%)	*Very good*, *n* (%)	*Excellent*, *n* (%)
1. Information you were given	698	20 (2.9)	37 (5.3)	218 (31.2)	312 (44.7)	111 (15.9)
2. Instructions	698	24 (3.4)	37 (5.3)	226 (32.4)	283 (40.5)	128 (18.3)
3. Ease of getting information	698	16 (2.3)	17 (2.4)	213 (30.5)	313 (44.8)	139 (19.9)
4. Information given by nurses	698	140 (20.1)	156 (22.3)	195 (27.9)	131 (18.8)	76 (10.9)
5. Informing family or friends	696	182 (26.1)	179 (25.7)	191 (27.4)	106 (15.2)	38 (5.5)
6. Involving family/friends in your care	696	183 (26.3)	178 (25.6)	181 (26.0)	97 (13.9)	57 (8.2)
7. Concern and caring by nurses	698	13 (1.9)	20 (2.9)	181 (25.9)	294 (42.1)	190 (27.2)
8. Attention of nurses to your condition	698	12 (1.7)	49 (7.0)	203 (29.1)	295 (42.3)	139 (19.9)
9. Recognition of your opinions	698	31 (4.4)	123 (17.6)	305 (43.7)	158 (22.6)	81 (11.6)
10. Consideration of your needs	698	16 (2.3)	74 (10.6)	297 (42.6)	202 (28.9)	109 (15.6)
11. The daily routine of the nurses	698	17 (2.4)	69 (9.9)	330 (47.3)	199 (28.5)	83 (11.9)
12. Helpfulness	698	9 (1.3)	33 (4.7)	192 (27.5)	322 (46.1)	142 (20.3)
13. Nursing staff response to your calls	698	20 (2.9)	42 (6.0)	252 (36.1)	270 (38.7)	114 (16.3)
14. Skill and competence of nurses	698	16 (2.3)	27 (3.9)	220 (31.5)	304 (43.6)	131 (18.8)
15. Coordination of care	698	13 (1.9)	23 (3.3)	227 (32.5)	318 (45.6)	117 (16.8)
16. Restful atmosphere provided by nurses	698	12 (1.7)	31 (4.4)	263 (37.7)	280 (40.1)	112 (16.0)
17. Privacy	698	1 (0.1)	10 (1.4)	255 (36.5)	314 (45.0)	118 (16.9)
18. Discharge instructions	696	424 (60.9)	36 (5.2)	97 (13.9)	91 (13.1)	48 (6.9)
19. Coordination of care after discharge	692	431 (62.3)	55 (7.9)	92 (13.3)	71 (10.3)	43 (6.2)

*Note:* All items were rated on a 5‐point Likert scale (1 = *poor*, 5 = *excellent*). Percentages are based on valid responses; missing data ranged from 0.3% to 0.9% across items.

**TABLE 3:** Distribution of responses for the four global satisfaction items (*N* = 698).

Item	*n*	*Poor*, *n* (%)	*Fair*, *n* (%)	*Good*, *n* (%)	*Very good*, *n* (%)	*Excellent*, *n* (%)
20. Overall quality of care and services	698	12 (1.7)	36 (5.2)	226 (32.4)	308 (44.1)	116 (16.6)
21. Overall quality of nursing care	698	13 (1.9)	40 (5.7)	218 (31.2)	296 (42.4)	131 (18.8)
22. Overall health self‐assessment	698	54 (7.7)	166 (23.8)	331 (47.4)	104 (14.9)	43 (6.2)
23. Recommendation of the hospital to family and friends	698	25 (3.6)	53 (7.6)	303 (43.4)	141 (20.2)	176 (25.2)

*Note:* Percentages are based on valid responses. No missing data were observed for these items.

### 3.3. EFA

In the derivation sample, EFA suggested the presence of a dominant first factor (first eigenvalue = 12.51; second eigenvalue = 2.23), followed by a marked drop in eigenvalues. Although the one‐factor solution showed uniformly high loadings, global fit was suboptimal (*χ* ^2^ (152) = 575.537, RMSEA = 0.089, CFI = 0.974, TLI = 0.971, SRMR = 0.237). The two‐factor solution provided substantially improved fit (*χ* ^2^ (134) = 308.897, RMSEA = 0.061, CFI = 0.989, TLI = 0.987, SRMR = 0.098) and yielded an interpretable structure. The first factor captured core nursing care during hospitalization (14 items), whereas the second factor encompassed items related to continuity of care, discharge information, and family involvement (5 items). The two factors were moderately correlated (*r* = 0.474).

### 3.4. CFA

In the validation sample, the unidimensional model showed acceptable but not optimal fit (*χ* ^2^ (152) = 647.112, RMSEA = 0.097 [0.089–0.105], CFI = 0.963, TLI = 0.959, SRMR = 0.175), with substantial residual misfit suggesting localized multidimensionality and indicating that a single latent factor did not fully capture the covariance structure of the items. The correlated two‐factor model demonstrated good fit (*χ* ^2^ (151) = 331.198, RMSEA = 0.059 [0.050–0.067], CFI = 0.987, TLI = 0.985, SRMR = 0.086). Standardized loadings ranged from 0.805 to 0.943 for the core nursing care factor and from 0.898 to 0.993 for the continuity/discharge factor. Item *R* ^2^ values were consistently high across both dimensions. The latent correlation between the two factors was moderate (*r* = 0.574), indicating related but distinguishable constructs.

A bifactor model specifying a general satisfaction factor and an orthogonal continuity/discharge‐specific factor was also tested. However, this model demonstrated inadequate fit (*χ* ^2^ (149) = 554.591, RMSEA = 0.089, CFI = 0.970, TLI = 0.966, SRMR = 0.101) and produced an inadmissible solution, with a nonpositive definite residual covariance matrix and a negative residual variance (Heywood case) involving Item i4. Given both statistical deterioration and numerical instability, the bifactor model was not retained.

In the total sample, the correlated two‐factor model also demonstrated acceptable fit (Figure 1) (*χ* ^2^ (151) = 427.988, RMSEA = 0.051 [0.046–0.057], CFI = 0.987, TLI = 0.985, SRMR = 0.080; Figure 1). Standardized loadings ranged from 0.818 to 0.953 for the core nursing care factor and from 0.872 to 0.987 for the continuity/discharge factor. Item *R* ^2^ values were consistently high across both dimensions. The latent correlation between the two factors was moderate (*r* = 0.562). Therefore, the correlated two‐factor model was selected as the final measurement structure. The first dimension was labeled “Core Nursing Care,” as it captures patients’ evaluations of the relational, communicative, and professional aspects of direct nursing care delivered during hospitalization. The second dimension was labeled “Continuity of Care,” as it encompasses patients’ perceptions of discharge planning, coordination of care, family involvement, and transitional processes extending beyond the immediate nurse–patient interaction.

**FIGURE 1:**
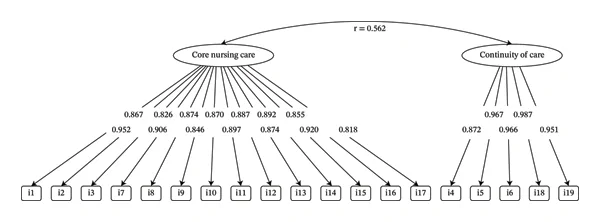
Confirmatory factor analysis of the Patient Satisfaction with Nursing Care Quality Questionnaire (PSNCQQ) in the total sample (*n* = 698). Note. Standardized factor loadings are shown; all were significant at *p* < 0.001. The curved arrow indicates the correlation between the two latent factors (*r* = 0.562). Model estimated using WLSMV with clustering adjustment; residuals omitted for clarity.

### 3.5. Reliability and Convergent Validity

Internal consistency was high for both latent dimensions identified in the correlated two‐factor model. For the core nursing care factor (14 items), McDonald’s omega was 0.964, indicating excellent reliability. For the continuity/discharge and family involvement factor (5 items), McDonald’s omega was 0.894. For the overall 19‐item scale, the internal consistency was also excellent (McDonald’s omega = 0.950). Cronbach’s alpha coefficients were consistent with these estimates (*α* = 0.95 for the total scale; *α* = 0.95 for core nursing care and *α* = 0.889 for continuity of care). CR exceeded the recommended threshold of 0.70 for both factors, further supporting internal consistency under a congeneric measurement framework. The AVE was greater than 0.50 for both dimensions, indicating that more than half of the variance in the respective indicators was explained by their underlying latent construct.

### 3.6. Convergent and Known‐Groups Validity

As shown in Table 4, the PSNCQQ total score demonstrated very strong correlations with overall perception of quality of care (*r* = 0.833, *p* < 0.001) and quality of nursing care (*r* = 0.829, *p* < 0.001), as well as a substantial association with likelihood of recommending the hospital (*r* = 0.629, *p* < 0.001). The correlation with perceived quality of health was moderate (*r* = 0.452, *p* < 0.001). The same pattern was observed for the two subscales, with the core nursing care dimension showing strong associations with global care evaluations, whereas the discharge/continuity dimension exhibited moderate but consistent correlations. Regarding known‐groups validity, gender was negatively associated with the total and both subscale scores (*r* range = −0.106 to −0.133, all *p* < 0.01), indicating slightly lower satisfaction among female patients. Educational level was positively associated with overall satisfaction and both dimensions, with a stronger effect observed for discharge/continuity (*r* = 0.241, *p* < 0.01). Previous hospitalizations were weakly associated only with the discharge dimension (*r* = 0.108, *p* < 0.01), while MMSE scores showed small positive associations with total and subscale scores.

**TABLE 4:** Correlations between PSNCQQ scores and external variables.

Variable	Total score	Core nursing care	Continuity of care
Overall perception of quality of care	0.833^∗∗^	0.877^∗∗^	0.510^∗∗^
Quality of nursing care	0.829^∗∗^	0.868^∗∗^	0.518^∗∗^
Perceived quality of health	0.452^∗∗^	0.388^∗∗^	0.446^∗∗^
Likelihood of recommending the hospital	0.629^∗∗^	0.626^∗∗^	0.461^∗∗^
Gender (female vs. male)	−0.133^∗∗^	−0.126^∗∗^	−0.106^∗∗^
Educational level (≤ 8 vs. > 8 years of education)	0.218^∗∗^	0.172^∗∗^	0.241^∗∗^
Previous hospitalizations (≥ 2 vs. 1)	0.045	0.007	0.108^∗∗^
Self‐rated health	0.024	−0.027	0.121^∗∗^
Mini‐Mental State	0.144^∗∗^	0.103^∗∗^	0.177^∗∗^

*Note:* Values represent Pearson correlation coefficients.

^∗^ *p* < 0.05 (two‐tailed).

^∗∗^ *p* < 0.01.

## 4. Discussion

The present study aimed to evaluate the psychometric properties of the Italian version of the PSNCQQ, a widely used instrument for assessing patient‐perceived quality of nursing care in hospital settings. To our knowledge, this is the first validation of the PSNCQQ conducted in Italy on a large multicenter sample of hospitalized adult patients. The findings indicate that the PSNCQQ is a valid, reliable, and conceptually coherent instrument suitable for use in clinical practice, research, and continuous quality improvement initiatives.

Descriptive analyses revealed high satisfaction levels with interpersonal and professional aspects of nursing care—such as empathy, helpfulness, and competence—confirming that relational and communicative dimensions are critical drivers of patient satisfaction [7, 16]. In contrast, lower scores were observed for items concerning discharge information and postdischarge coordination. These findings echo international evidence identifying the transition from hospital to home as one of the most vulnerable phases in the care continuum [38], a finding confirmed by more recent evidence on hospital‐to‐home transitions [39, 40]. Poor discharge communication and insufficient continuity of care undermine patient safety, increase readmission risk, and reflect systemic fragmentation between acute and community services.

Within the broader framework of patient‐centered care, as defined by the Institute of Medicine Committee on Quality of Health Care in America [41], these findings highlight the need to place the patient’s perspective at the core of care design and evaluation. Patient satisfaction is not merely an outcome indicator but a proxy for the extent to which care processes respect individual preferences, needs, and values. As Donabedian [42] posited, quality of care arises from the interplay between structure, process, and outcome; patient satisfaction represents an outcome that both reflects and influences the quality‐of‐care processes. The PSNCQQ thus offers a practical, theory‐consistent measure for operationalizing patient‐centered quality assessment in nursing.

CFA did not fully support the strictly unidimensional structure originally proposed by Laschinger et al. [22]. Although a dominant general dimension of satisfaction was evident, the data were better represented by a correlated two‐factor model distinguishing core nursing care from continuity/discharge‐related aspects. The emergence of two factors in the Italian context may reflect organizational and cultural features of hospital care delivery. In particular, discharge planning, family involvement, and continuity of care often involve structured procedural elements and interprofessional coordination that are organizationally distinct from bedside nursing interactions. In health systems such as Italy’s, where discharge processes may vary substantially across hospitals and are embedded within regional administrative frameworks, patients may differentiate between relational aspects of nursing care and transitional care processes [43]. Additionally, the decision to adapt Item 5 during the content validity phase strengthens the cultural and ethical relevance of the Italian PSNCQQ. The original wording referred to nurses informing family members or friends about the patient’s condition. However, in the Italian and broader European regulatory context, such communication is governed by legal and ethical frameworks that require explicit patient consent (GDPR 2016/679; FNOPI Code of Ethics, 2019). The revised item, focused on family involvement in care planning rather than information disclosure, aligns with ethical nursing practice and patient rights legislation while preserving the original construct domain. This adaptation reflects best practices in cross‐cultural validation, ensuring linguistic accuracy as well as conceptual, normative, and cultural equivalence.

The bidimensional solution retained here can be read against the evidence produced by earlier psychometric studies of the instrument. The original Canadian validation reported a single factor with loadings between 0.75 and 0.89 [22], and unidimensionality was subsequently replicated in the Serbian [44] and in the Croatian version, the latter accounting for 70.64% of the total variance with a Cronbach’s alpha of 0.977 [45]. The Arabic adaptation is the only previous version reporting two factors, separating satisfaction with the care provided from satisfaction with the information provided and explaining 69.3% of the variance; that solution, however, was reached after removing the two items on discharge instructions and postdischarge coordination, resulting in a 17‐item instrument [5]. Our results are therefore only partially consistent with the prevailing unidimensional evidence and come closer to the Arabic adaptation, with the substantive difference that the two discharge‐related items were retained and, together with the items on family involvement, constitute the second dimension. Rather than a psychometric artifact, this pattern is coherent with the pronounced floor effects observed for those same items and suggests that, where discharge planning and postdischarge coordination are organizationally weak or highly variable across facilities, patients appraise them separately from bedside nursing care. Direct comparability of the factor structure across language versions should nonetheless be established through measurement invariance testing rather than inferred from separate validations.

Internal consistency reliability was excellent for both latent dimensions and for the total score, confirming the robustness of the PSNCQQ in measuring patient satisfaction within the Italian context. These findings are consistent with previous international studies reporting high reliability across linguistic versions of the instrument [5, 19, 44]. The magnitude of the item–total correlations and the high omega coefficients indicate that items within each domain contribute meaningfully to their respective constructs, while the elevated reliability of the total score suggests the presence of a strong overarching satisfaction dimension. The use of complementary reliability indices, including McDonald’s omega, strengthens the evaluation under a congeneric measurement framework and aligns with contemporary psychometric recommendations [46]. Importantly, the high reliability observed across domains supports the interpretability of both subscale and total scores, indicating that observed differences primarily reflect true variability in perceived satisfaction rather than measurement error.

Convergent validity was supported by adequate AVE and CR indices for both latent dimensions, indicating that the variance explained by each factor exceeded measurement error. Construct validity was further demonstrated through strong and theoretically coherent associations between PSNCQQ scores and global satisfaction indicators. The total score showed very strong correlations with overall perception of care quality and quality of nursing care and substantial associations with willingness to recommend the hospital. These findings align with theoretical expectations and prior evidence suggesting that satisfaction with nursing care is a central determinant of overall hospital satisfaction [17, 47]. One expected correlation was that female patients reported slightly lower satisfaction levels. This finding may reflect higher expectations and greater involvement in healthcare processes, as women are more frequently engaged both as patients and informal caregivers [48]. Greater familiarity with healthcare services and heightened sensitivity to relational and communicative aspects of care may contribute to more critical evaluations when expectations are not fully met [49]. A small but significant positive association was observed between MMSE scores and satisfaction, particularly for the continuity of care dimension. Patients with higher cognitive functioning may better comprehend discharge instructions, perceive coordination as more coherent, and experience greater confidence in care transitions, thereby reporting higher satisfaction.

### 4.1. Strengths and Implications

A major strength of this study is the large multicenter sample, encompassing diverse hospital settings and clinical wards, which enhances heterogeneity and generalizability. Structural validity was strengthened through a split‐sample design, with EFA and CFA conducted on independent subsamples, reducing the risk of overfitting and improving replicability. Analyses also accounted for clustering by hospital, further supporting the robustness of statistical inferences. From a clinical and organizational perspective, the findings offer actionable evidence for integrating patient feedback into continuous quality improvement systems. The PSNCQQ can support hospital governance in monitoring nursing‐sensitive indicators and evaluating organizational initiatives. The strong association between satisfaction and relational aspects of care highlights the importance of investing in nurses’ communication and educational competencies, while targeted interventions in discharge planning and interdisciplinary coordination may improve continuity‐related satisfaction.

### 4.2. Limitations

This study has several limitations that should be acknowledged. Its cross‐sectional design precludes assessment of test–retest reliability and temporal stability. All measures were self‐reported, potentially introducing social desirability or recall bias. Additionally, although adjustments were made for clustering, the number of clusters was small (five hospitals), which is below the number usually recommended for cluster‐robust estimation; the standard errors reported here should therefore be interpreted with caution, and future research using multilevel CFA on a larger number of sites could provide deeper insights into within‐ and between‐hospital variance components. The study also relied primarily on correlations with global satisfaction items for construct validation; future investigations could extend validation to external constructs such as perceived safety, nurse communication quality, or trust in the healthcare team. Despite these limitations, the study provides robust initial evidence for the validity and reliability of the PSNCQQ.

### 4.3. Future Research and Implications for Practice

Future research should examine measurement invariance across demographic and clinical subgroups and assess the responsiveness of the PSNCQQ to quality improvement interventions. Longitudinal designs could clarify whether variations in PSNCQQ scores correspond to measurable changes in care processes and patient outcomes. Integration of the instrument into electronic health records or hospital dashboards may enable real‐time monitoring and support data‐driven decision‐making.

From a practice perspective, the systematic use of validated patient‐reported experience measures aligns with the shift toward value‐based and patient‐centered healthcare [50]. The bidimensional structure identified in this study suggests that improvement strategies should address both relational aspects of bedside nursing care and organizational processes related to discharge and continuity. Regular feedback can inform reflective practice, strengthen nursing‐sensitive indicators, and enhance the visibility of nursing contributions within multidisciplinary teams.

This study also has implications for nursing education. Patient satisfaction reflects not only technical competence but also relational and communicative skills developed during professional training. Evidence indicates that empathy, clarity of information, collaboration, and continuity of care are central determinants of patient experience [51, 52]. Importantly, these dimensions are also strongly influenced by the presence and performance of nursing students during clinical placements, as highlighted by a recent systematic review [53]. Therefore, curricula in nursing education should integrate training strategies aimed at developing not only technical competencies but also soft skills such as patient‐centered communication, shared decision‐making, and professional caring. Embedding systematic patient feedback—using validated instruments such as the PSNCQQ—into clinical learning environments may support reflective practice and contribute to the development of high‐quality, person‐centered nursing care.

## Author Contributions

Donatella D’Accolti: conceptualization, methodology, project administration, writing–original draft, and writing–review and editing. Paolo Iovino: formal analysis, data curation, validation, writing–original draft, and writing–review and editing. Cinzia Anna Maria Papappicco: investigation, data collection, resources, and writing–review and editing. Sipontina Zerulo: methodology, statistical analysis, validation, and writing–review and editing. Federico Ruta: conceptualization, methodology, supervision, project administration, and writing–original draft, and writing–review and editing.

## Funding

No specific funding was received for this study.

Open‐access publishing was facilitated by Universita degli Studi di Firenze, as part of the Wiley ‐ CRUI‐CARE agreement.

## Disclosure

All authors approved the final manuscript and agreed to be accountable for all aspects of the work.

## Ethics Statement

The study adhered to the Declaration of Helsinki and was approved by the Independent Ethics Committee of University Hospital of Bari (Protocol No. 38977/20/04/23).

## Conflicts of Interest

The authors declare no conflicts of interest.

## Supporting Information

Additional supporting information can be found online in the Supporting Information section.

## Supporting information


**Supporting Information** Supporting Information S1. COSMIN‐based methodological appraisal of the PSNCQQ—validation study. This file reports the ratings and supporting justifications for each applicable COSMIN standard, together with an overall graphical summary.

## Data Availability

The datasets generated and/or analyzed during the current study are available from the corresponding author upon reasonable request.
