# Supplementary material for: Italian Validation of the Patient Satisfaction With Nursing Care Quality Questionnaire (PSNCQQ): A Multicenter Psychometric Study
Source: J Nurs Manag. 2026 Oct 8;2026:7681817. doi: 10.1155/jonm/7681817 (PMC13650251; doi:10.1155/jonm/7681817)
Supplement: Supplementary file 1 [file JONM-2026-7681817-s001.pdf]

Supplementary Material S1. COSMIN-based methodological appraisal of the PSNCQQ-It validation study. This file reports the ratings and supporting justifications for each applicable COSMIN standard, together with an overall graphical summary.

SECTION 1: GENERAL STUDY CHARACTERISTICS

Standard	Rating	Justification
PROM name and version	**Very good**	Italian version of the Patient Satisfaction with Nursing Care Quality Questionnaire (PSNCQQ-It), 19 items on a 5-point Likert scale plus 4 global items are clearly described, including structure and scoring.
Construct	**Very good**	Construct defined as 'patient satisfaction with the quality of nursing care during hospitalization' and framed as a key nursing-sensitive quality indicator with theoretical and empirical background.
Development of original PROM	**Adequate**	Original PSNCQQ development is summarized and the primary validation study is cited, but full details are not reproduced in the current manuscript.
Context of use	**Very good**	Intended use (clinical quality assessment, governance, benchmarking and research) and setting (adult inpatients in medical and surgical wards of 5 Italian public hospitals) are explicitly stated.
Target population and sample	**Very good**	Adults ≥18 years, hospitalized ≥48 hours, Italian-speaking, cognitively intact (MMSE >25) and without delirium (CAM negative), consecutively recruited in 5 hospitals; N=698.

SECTION 2: CONTENT VALIDITY

Standard	Rating	Justification
Patients' content validity study	**Very good**	Cognitive debriefing interviews with 15 hospitalized patients using a think-aloud method to assess clarity, comprehensibility and relevance of all items.
Professionals' content validity study	**Very good**	Expert panel of 7 senior nurses and nursing professors evaluated semantic, conceptual and cultural equivalence and item relevance using a structured CVI-based grid.
Analysis of content validity	**Adequate**	Feedback from experts and patients was analyzed by at least two researchers and used to refine items (e.g. adaptation of Item 5 to GDPR and ethical norms), although analytic procedures are not extensively detailed.

SECTION 3: TRANSLATION PROCESS

Standard	Rating	Justification
Forward translation	**Very good**	Two independent bilingual translators conducted forward translations and a reconciled version was produced, following international guidelines.

SECTION 3: TRANSLATION PROCESS		
**Standard**	**Rating**	**Justification**
Back-translation	**Very good**	Two native English speakers, blinded to the original questionnaire, produced back-translations to verify semantic and conceptual equivalence.
Expert committee review	**Very good**	A multidisciplinary committee of 7 nurses/professors reviewed all versions, resolved discrepancies and approved the final Italian wording.
Pretesting / pilot testing	**Very good**	Cognitive debriefing with 15 hospitalized patients confirmed clarity, acceptability and cultural appropriateness of the final Italian version.

SECTION 4: STRUCTURAL VALIDITY		
**Standard**	**Rating**	**Justification**
Model specification for structural validity	**Very good**	A reflective unidimensional CFA model was specified to capture a single latent factor 'satisfaction with quality of nursing care', consistent with the original PSNCQQ conceptualization.
Statistical methods for structural validity	**Very good**	CFA using WLSMV for ordinal items and TYPE=COMPLEX to account for clustering by hospital; model fit evaluated with χ^2, RMSEA, CFI, TLI and SRMR and predefined cut-offs.
Sample size for structural validity	**Very good**	N=698, exceeding COSMIN recommendations (≥7 participants per item and ≥100 participants).
Handling of missing data (structural validity)	**Adequate**	Data entry with double-checking; missing data were monitored and handled according to predefined rules, with low overall missingness, though analytic handling is briefly described.

SECTION 5: INTERNAL CONSISTENCY		
**Standard**	**Rating**	**Justification**
Evidence of unidimensionality	**Very good**	CFA with good global fit after freeing two theoretically justified residual covariances, plus a bifactor/testlet model confirming an essentially unidimensional structure.
Sample size for internal consistency	**Very good**	Reliability indices estimated on N≈698 patients.
Internal consistency statistics	**Very good**	Cronbach's alpha=0.95, McDonald's omega=0.95, split-half coefficients ≈0.93, corrected item–total correlations 0.59–0.81 indicate excellent internal consistency.

SECTION 6: CONSTRUCT VALIDITY (HYPOTHESES TESTING)		
**Standard**	**Rating**	**Justification**
Hypotheses for convergent construct validity	**Very good**	Explicit a priori hypotheses of strong positive correlations between PSNCQQ total score and four global items (overall care, nursing care, health, recommendation). Comparator constructs are clearly described.
Methods for convergent construct validity	**Very good**	Correlation analyses with simultaneous administration of PSNCQQ and global items in the same sample; statistical methods are appropriate for the hypotheses.
Known-groups validity hypotheses	**Adequate**	Expectations regarding differences by age, sex, room type and occupational status are described and tested, though hypotheses are less formally specified than for global items.

SECTION 7: PROPERTIES NOT ASSESSED IN THIS STUDY		
**Standard**	**Rating**	**Justification**
Test–retest reliability	**NA**	No second administration was planned; test–retest reliability and ICCs were not assessed and are acknowledged as a limitation.
Measurement error (SEM, SDC, LoA)	**NA**	Not assessed because no repeated measurements were performed.
Responsiveness (longitudinal validity)	**NA**	Not assessed; the study has a cross-sectional design without follow-up or intervention.
Criterion validity	**NA**	Not applicable; no true gold standard exists for patient satisfaction PROMs, and no long-versus-short-form comparison was conducted.
Cross-cultural validity / measurement invariance	**NA**	Not assessed; only the Italian version was evaluated, without multi-language or multi-group invariance testing.
